# The relationship between serum Endocan and ADMA levels and penile Doppler ultrasonography findings in patients with severe erectile dysfunction

**DOI:** 10.1097/MD.0000000000041742

**Published:** 2025-03-14

**Authors:** Doruk Demirel, Binhan Kagan Aktas, Arzu Kosem, Turker Soydas, Cagri Akpinar, Emrah Gökay Ozgur, Cuneyt Ozden, Onder Kayigil

**Affiliations:** a Department of Urology, Ankara Etlik City Hospital, Ankara, Turkey; b Department of Urology, Ankara Bilkent City Hospital, Ankara, Turkey; c Department of Biochemistry, Ankara Etlik City Hospital, Ankara, Turkey; d Department of Biostatistics, Faculty of Medicine, Marmara University, Istanbul, Turkey.

**Keywords:** ADMA, Endocan, erectile dysfunction, penile Doppler

## Abstract

To evaluate the relationship between serum endothelial cell specific molecule-1 (Endocan), asymmetric dimethylarginine (ADMA) values, and penile Doppler ultrasonography (USG) findings in patients with severe erectile dysfunction (ED). This prospective study included 73 patients who were classified as severe ED and had an indication for penile Doppler USG in our urology outpatient clinic between April 2017 and January 2020. Fasting blood sugar, lipid profile, thyroid function tests, total testosterone, and serum Endocan and ADMA values were sampled, and penile Doppler USG examination data were recorded. Vasculogenic ED was detected in 51 (69.86%) of 73 patients, while the flow rates were normal in 22 (30.14%). Among those with vasculogenic ED, 15 (29.41%) had arterial insufficiency, 22 (43.13%) had venous leakage, and 14 (27.46%) had mixed. There was no statistically significant difference between the mean ADMA and Endocan values of ED with normal flow (14.44 ± 6.20 ng/mL and 0.18 ± 0.14 ng/mL) and the vasculogenic ED (12.31 ± 5.86 ng/mL and 0.21 ± 0.16 ng/mL) groups (*P* = .097 for ADMA and *P* = .315 for Endocan, respectively). Endocan and ADMA levels were not predictors of severe ED. There is a need for multicenter studies with larger patient populations to be conducted with different biomarkers that may have a higher predictive value in the future.

## 1. Introduction

Erectile dysfunction (ED) is defined as the failure to achieve and/or maintain penile erection at the level required for satisfactory sexual performance.^[1]^ ED severity was rated using the International Index of Erectile Function form.^[2]^ An abridged and simplified 5-item version of the International Index of Erectile Function (IIEF-5) is more frequently used as a diagnostic tool for ED. Severe ED was evaluated as 5–7 points in the IIEF-5.^[3]^ The etiology of ED includes psychogenic and mostly organic causes, and these may be of vasculogenic, neurogenic, anatomical and endocrine origin.^[4]^ Vasculogenic ED accounts for most organic ED and may be due to abnormalities in penile arterial inflow and/or venous outflow. Vasculogenic ED is by far the most common etiology of organic ED. Indeed, ED can be a manifestation of an underlying vascular disorder.^[5]^ Importantly, ED is no longer simply confined to sexual activities but acts as an indicator of systemic endothelial dysfunction.^[6]^ Therefore, studies on some biomarkers reflecting endothelial functions have gained importance.

Endothelial cell-specific molecule-1 (Endocan) is a sulfated glycoprotein released from the endothelium. It is involved in events in the endothelium, such as tumoral adhesion, progression, migration, angiogenesis and inflammatory diseases. Endocan regulates leukocyte migration by increasing the release of proinflammatory cytokines from endothelial cells and increasing microvascular permeability. It also causes neointima formation in atherosclerosis by stimulating the proliferation and migration of vascular smooth muscle cells. Its wide role in endothelial-mediated pathologies makes the Endocan molecule an important biomarker in all conditions that cause endothelial dysfunction, from cardiovascular events to cancer.^[7–9]^

Asymmetric dimethyl arginine (ADMA) is a nitric oxide (NO) synthase inhibitor proteoglycan formed by intracellular methylation of the amino acid L-arginine. ADMA is valuable in predicting cardiovascular disease (CVD) risk as a biomarker associated with endothelial dysfunction.^[10]^ The mechanism of endothelial dysfunction caused by ADMA is explained as an increase in the amount of superoxide due to a decrease in vascular NO formation.^[11]^ Penile Doppler ultrasound (USG) is a valuable minimally invasive examination in the vascular evaluation of ED.^[12]^ It requires intra-cavernosal vasoactive agent injection, which exposes patients to the risk of developing severe complications, especially priapism.^[13]^

In this study, the relationship between serum endothelial biomarkers and penile Doppler USG findings was investigated. Our possibility of diagnosing vasculogenic ED by blood tests without performing USG has been questioned.

## 2. Materials and methods

**Figure 1.**
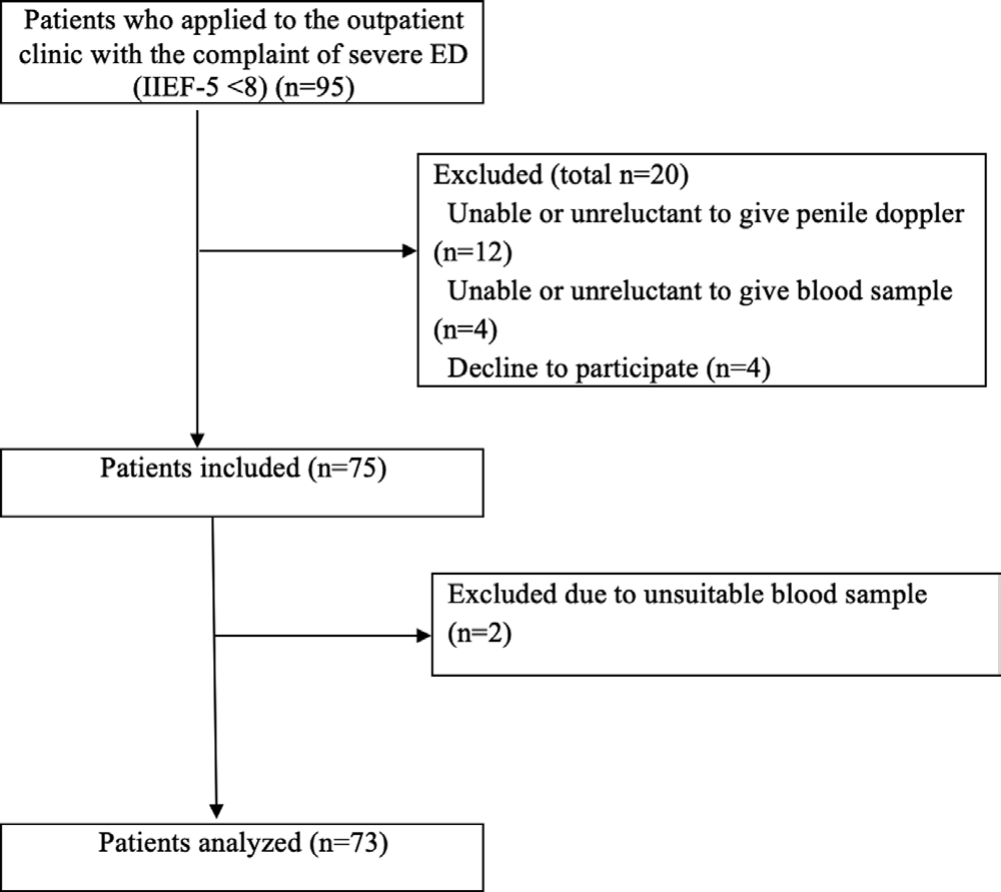
Flow chart illustrating the patient selection process. ED = erectile dysfunction, IIEF-5 = 5-item International Index of Erectile Function.

This prospective non-randomized experimental clinical study was started on April 2017 in Ankara Numune Training and Research Hospital and finished in January 2020 in Ankara Bilkent City Hospital in Turkiye. All procedures performed in studies involving human participants were in accordance with the ethical standards of the institutional and/or national research committee at which the studies were conducted (approval number; E-18-23-52) and with the 1964 Helsinki Declaration and its later amendments or comparable ethical standards. This article does not contain any studies with animals performed by any of the authors. All participants have given informed consent before inclusion in the present study. Patients evaluated as severe ED according to the IIEF-5 form were included in this study. The total score varies from 1 to 25 points. It was evaluated as 5 to 7 points severe ED, 8 to 11 points as moderate ED, 12 to 16 points as mild moderate ED, 17 to 21 points as mild ED and 22 to 25 points as no ED.^[3]^ In the anamnesis taken from the patients, the duration of ED, presence of morning stiffness, smoking, presence of hypertension (HT), diabetes mellitus (DM) or CVD, age of the patients, IIEF-5 score, and whether they used phosphodiesterase-5 enzyme inhibitor drugs due to ED were noted. All in all, the first 95 patients presenting with severe ED were registered to this study. The flow chart of the participants with reasons for exclusion is provided in Figure 1. Those who had previous pelvic trauma or surgery, patients who had benefited from using phosphodiesterase-5 inhibitors, those with psychogenic ED and moderate or mild ED according to IIEF-5, endocrine and neurological disease, chronic liver disease and patients with chronic renal failure and those who used drugs that could cause ED were excluded from this study.

Fasting blood sugar, lipid profile (total cholesterol, triglyceride, low density and high-density lipoproteins), thyroid function tests (free T3 and T4, thyroid stimulating hormone), total testosterone, and additionally serum Endocan and ADMA values were sampled and penile Doppler USG examination data were recorded. According to penile Doppler USG results, ED patients were divided into 4 groups: arterial insufficiency (AI), venous leakage (VL), both arterial insufficiency and venous leakage (mixed [M]) and ED with normal flow (NF).

### 2.1. Laboratory tests

The patients’ blood was taken between 08:00 and 09:30 in the morning after 12 hours of fasting. An extra tube of blood for Endocan and ADMA was taken, sent to the central biochemistry laboratory and kept at −40°C until the kits (BT Lab brand Human Endothelial Cell-specific Molecule-1 ELISA kit with product code E3160HU [96 tests] and BT Lab brand Human Asymmetrical Dimethylarginine ELISA kit with product code E1887HU [96 tests]) are available.

### 2.2. Penile Doppler USG measurements

Before the procedure, all patients were informed about how penile Doppler USG was performed and the complications they might encounter. Pain, bleeding, bruising in the procedure area and priapism were explained. In case of priapism, emergency intervention will be required within 4 hours after the application. Just before the procedure, an ampoule (50 mg) of intra-cavernosal papaverine was applied to the patients. Erection hardness was assesed according to 5-point response score based on the inspection of the patients’ erection.^[14]^ Arterial and venous flow rates per 5, 10, 15, 20, 25 minutes were evaluated.

According to penile Doppler USG results, patients with peak systolic velocity (PSV) < 30 cm/second were grouped as AI, end diastolic velocity (EDV) > 5 cm/second as VL, and both PSV < 30 cm/second and EDV > 5 cm/second as M. Patients with PSV > 30 cm/second and EDV < 5 cm/second were also included in the ED with NF group. The resistive index (RI) was also evaluated in patients with VL. RI is a parameter that shows veno-occlusive disease in patients with normal arterial flow rates but found to have VL and was calculated with the (PSV-EDV)/PSV formula. RI values above 0.8 were considered significant for veno-occlusion.^[15]^

### 2.3. Statistical analysis

The minimum sample size was calculated as 66 using the G power software program (latest version 3.1.9.7; Heinrich Heine Universität Düsseldorf, Düsseldorf, Germany) IBM SPSS® Statistics version 25 (IBM Corp., Armonk) was used for statistical analysis. The normal distribution of the continuous variables was tested using the Kolmogorov–Smirnov test. The mean and standard deviations of all evaluated parameters were noted. For independent variables non-normally distribution, Mann–Whitney *U* test was used for statistical comparison of 2 groups, and Kruskal–Wallis test was used to compare more than 2 groups. Receiver operating characteristic curve analyses were performed for predictive accuracy with the area under the receiver operating curve. *P* values of < .05 in the 95% confidence interval were considered statistically significant.

## 3. Results

A sum of 22 patients had severe ED with NF, of vasculogenic ED patients, 15 had AI, 22 had VL and 14 had M pathology. The mean age was 48.27 ± 9.43 years in AI group, 49.23 ± 10.13 years in VL group, 57.71 ± 13.45 years in M group and 45.27 ± 11.86 years in ED with NF group. A sum of 46 of 73 patients were smokers. A sum of 21 patients had HT, 20 had DM, and 13 had CVD. A significant association with severe ED was observed only in patients with CVD.

Table 1 compares the Endocan and ADMA values of patients with NF and vasculogenic ED. There was no statistically significant difference regarding ADMA and Endocan values between patients with NF and those with vasculogenic ED (*P* = .097 for ADMA, *P* = .315 for Endocan). There was no statistically significant difference regarding ADMA and Endocan values between ED with NF and vasculogenic ED subgroups (AI, VL, M; *P* = .233 for ADMA, *P* = .540 for Endocan). There was a significant difference between normal flow ED patients and the other 3 vasculogenic subgroups regarding the presence of (CVD) (Table 2). When the ADMA and Endocan values of the 3 vasculogenic ED subgroups and the normal flow ED group were compared one by one with the remaining patients, no significant difference was detected (Table 3). As a result of the receiver operating characteristic analysis for ADMA and Endocan values, the *P* value was not significant so that no predictive value could be obtained (for ADMA *P* = .097, for Endocan *P* = .315).

**Table 1:** Comparison of ADMA and Endocan values of ED with NF and vasculogenic ED patients.

Group		ADMA (ng/mL)	Endocan (ng/mL)
ED with NF	N	22	
	Mean	14.4360	0.1839
	Median	13.7442	0.1264
	Standard deviation	6.19611	0.13637
	Minimum	7.82	0.06
	Maximum	30.31	0.60
Vasculogenic ED	N	51	
	Mean	12.3056	0.2147
	Median	10.5394	0.1675
	Standard deviation	5.85579	0.15884
	Minimum	7.80	0.01
	Maximum	35.43	0.74
*P* value		.097	.315

ADMA = asymmetric dimethylarginine, ED = erectile dysfunction, NF = normal flow.

**Table 2:** Comparison of ADMA and Endocan values and other data between ED with NF and vasculogenic ED subgroups (AI, VL, M).

Parameters	AI (N = 15)	VL (N = 22)	M (N = 14)	NF (N = 22)	*P* value
Age (yr)	48.27 ± 9.43	49.23 ± 10.13	57.71 ± 13.45	45.27 ± 11.86	.283*
HDL (mg/dL)	42.6 ± 9.73	39.32 ± 5.22	44 ± 8.73	45 ± 10.56	.171*
TTEST (mg/dL)	4.02 ± 2.01	4.12 ± 1.30	3.66 ± 1.13	4.60 ± 1.95	.423*
LDL (mg/dL)	96 (80–140)	106 (95.75–120.75)	91 (61.25–127.50)	98.00 (82.75–115.75)	.509**
TG (mg/dL)	147 (97–204)	164 (118–239.25)	172.50 (125.50–204.50)	157 (89.75–186.50)	.582**
TCHOL (mg/dL)	180.53 (156.25–186.50)	176 (163.50–205.25)	168.50 (123–210)	177 (156.25–186.50)	.712**
FBS (mg/dL)	102 (91–218)	98.50 (88.50–150.25)	95 (90–107.75)	91 (84.75–109)	.202**
T3 (ng/L)	3.39 (2.56–3.72)	3.37 (2.49–3.56)	2.36 (1.21–3.41)	3.32 (3.11–3.72)	.316**
T4 (ng/L)	1.23 (1.12–1.47)	1.20 (1.10–1.40)	1.31 (1.03–1.46)	1.25 (1.13–1.60)	.857**
TSH (mU/L)	1.42 (0.97–1.90)	1.12 (0.85–2.31)	1.25 (0.75–2.86)	1.53 (1.15–2.07)	.548**
IIEF-5	8 (7–8)	8 (5.75–9)	6.00 (4–7.50)	8 (6.75–8)	.071**
Mean PS	19.50 (15.41–22.00)	39.75 (36.12–45)	27.75 (19.37–31.87)	44.25 (38.50–53.82)	<.001**
Mean END	1.54 (0–2.50)	10 (8–13.62)	7.50 (5.56–9.72)	0 (0–2.88)	<.001**
ADMA (ng/mL)	10.99 (9–16.03)	11.13 (8.45–15.02)	10.20 (8.41–10.91)	13.74 (8.95–17.16)	.233**
Endocan (ng/mL)	0.16 (0.11–0.25)	0.15 (0.10–0.30)	0.22 (0.11–0.31)	0.12 (0.09–0.26)	.540**
Smoker					
No	4 (26.7)	7 (31.8)	5 (35.7)	11 (50.0)	.467***
Yes	11 (73.3)	15 (68.2)	9 (64.3)	11 (50.0)	
HT					
No	11 (73.3)	15 (68.2)	9 (64.3)	17 (77.3)	.862***
Yes	4 (26.7)	7 (31.8)	5 (35.7)	5 (22.7)	
DM					
No	10 (66.7)	14 (63.6)	10 (71.4)	19 (86.4)	.332***
Yes	5 (33.3)	8 (36.4)	4 (28.6)	3 (13,6)	
CVD					
No	10 (66.7)	20 (90.9)	9 (64.3)	21 (95.5)	.021***
Yes	5 (33.3)	2 (9.1)	5 (35.7)	1 (4.5)	

ADMA = asymmetric dimethylarginine, AI = arterial insufficiency, CVD = cardiovascular disease, DM = diabetes mellitus, ED = erectile dysfunction, END = end diastolic, FBS = fasting blood sugar, HDL = high density lipoprotein, HT = hypertension, IIEF-5 = The International Index of Erectile Function, LDL = low density lipoprotein, M = mixed, NF = normal flow, PS = peak systolic, SD = standard deviation, TCHOL = total cholesterol, TG = triglyceride, TSH = thyroid stimulating hormone, TTEST = total testosterone, VL = venous leakage.

* Independent sample groups *t* test (mean ± SD).

** Mann–Whitney *U* test (median [IQR]).

*** Chi-square test (n [%]).

**Table 3:** Comparison of ADMA and Endocan values of the 3 vasculogenic ED subgroups (AI, VL, M) and ED with NF group.

Group	ADMA (ng/mL)	*P* value	Endocan (ng/mL)	*P* value
AI		.924		.967
Yes	11.07 (8.52–15.50)		0.15 (0.10–0.27)	
No	10.99 (9.00–16.03)		0.16 (0.11–0.25)	
VL		.764		.914
Yes	10.99 (8.65–15.91)		0.16 (0.10–0.26)	
No	11.13 (8.45–15.02)		0.15 (0.10–0.30)	
M		.093		.181
Yes	11.35 (8.54–16.03)		0.15 (0.10–0.26)	
No	10.20 (8.41–10.91)		0.22 (0.11–0.31)	
ED with NF		.097		.315
Yes	10.53 (8.48–14.25)		0.16 (0.11–0.29)	
No	13.74 (8.95–17.16)		0.12 (0.09–0.26)	

ADMA = asymmetric dimethylarginine, AI = arterial insufficiency, ED = erectile dysfunction, M = mixed, NF = normal flow, VL = venous leakage.

* “Mann–Whitney *U”* test (Median [Q1–Q3]).

## 4. Discussion

Endothelial dysfunction is an inflammatory process characterized by disruption in NO pathways and consequent problems in the vasodilation mechanism before the development of atherosclerotic plaque. Therefore, a significant pathophysiological relationship between ED and CVD can be mentioned.^[15,16]^ Loss of NO in endothelial cells has been reported in the early stage of atherosclerosis. Also, it has been reported that low NO is also associated with other atherogenic risk factors, such as dyslipidemia, HT, DM, age, smoking, hyperhomocysteinemia, menopause, and family history.^[17,18]^ In a study by Morley et al,^[19]^ 57% of men who had coronary bypass surgery and 64% of men who had myocardial ischemia (MI) had ED. A 2-year randomized, controlled trial investigating whether ED is a predictor of cardiovascular events has also shown that ED is a strong predictor of MI, stroke, and heart failure in those with pre-existing CVD.^[20]^ Similarly, a significant association was shown between CVD and severe ED in our study.

It has been emphasized in some studies that biomarkers, such as high-sensitivity CRP, hemoglobin A1c, urinary albumin excretion (microalbuminuria) and lipoprotein-related phospholipase A2 in serum can be used as an aid in demonstrating vascular risk in asymptomatic patients. It has been stated that these markers are positively correlated with ED and also with CVD.^[21,22]^ Kandeel et al^[23]^ stated that serum fetuin-A glycoprotein showed a positive correlation with IIEF-5 and could be used to predict ED. In a study conducted by Ciftci et al^[21]^ in 2007, the role of paraoxonase enzyme in ED and atherosclerosis was investigated. It was shown that paraoxonase activity was significantly lower in patients with ED. In our current study, we evaluated serum Endocan and ADMA levels in patients with severe ED, since various biomarkers have been studied and significant results have been obtained in predicting ED, and that these markers also play a role in other vascular pathologies, such as CVD.

Karabakan et al and Onuk et al, in which they evaluated the Endocan levels and the severity of ED, a significant difference was found between the plasma Endocan levels between the severe ED group and the control group.^[8,9]^ Penile Doppler USG was not applied to patients with ED in both studies. In the study of Akarsu et al, penile Doppler USG was performed in mild, moderate and severe ED patients. A significant correlation was found between PSV, an indicator of AI, and serum Endocan levels.^[24]^ In our study, no significant correlation was found between PSV and serum Endocan and ADMA values between our groups. The reason for this may be that there were no patients with mild or moderate ED in our study, all of them were patients with severe ED, and these patients were not only with isolated AI, but also included VL and M pathologies.

ADMA is a NO synthase enzyme inhibitor, and as serum levels increase, NO synthesis decreases. It has been reported that ADMA plays a role in endothelial dysfunction and atherosclerotic processes and may be a biomarker.^[25]^ As mentioned earlier, endothelial dysfunction indicates the early stage of atherosclerosis, and ADMA is an important molecule in predicting CVD risk as a marker of endothelial dysfunction.^[10]^ Richter et al and Sydow et al reported that just like Endocan, ADMA levels increase in HT, CVD, DM, chronic renal disease and heart failure, and endothelial dysfunction caused by ADMA is associated with the inhibition of vascular NO synthesis and increased superoxide levels.^[26–28]^ In our study, no statistically significant difference was found between our groups regarding serum ADMA values.

In our study, penile Doppler USG was applied to 73 patients who fit the severe ED classification using the IIEF-5 questionnaire. Penile Doppler USG revealed vascular pathology in 51 patients and normal flow in 22. Besides the psychogenic etiology, there may be another reason for this situation. While endothelial damage occurs first in ED patients, deterioration in flow rate may occur in later stages and vasculogenic ED may be seen in these patients after all. In other words, while endothelial damage occurs at a certain level in all severe ED patients, this may not have reflected its effect on flow rate yet. This may be the reason why the endothelial damage biomarkers evaluated in our study were not statistically different between these 2 groups with abnormal and normal flow rates. The fact that ED is a precursor to CVD also supports this theory. There are different applications in the literature regarding vasoactive agents, drug doses, and whether additional drug use is required. Also, it has been emphasized that there are attempts to standardize penile Doppler USG technique that is, the timing of measurements taken while applying penile Doppler USG and the variation in PSV and EDV cutoff values.^[29]^ In a previous study, it was stated that the probability of cardiovascular events increased in patients with PSV <25 cm/sn.^[30]^ In our study, standardization was tried to be achieved by performing penile Doppler USG with the same device and the same radiology team and administering the same drug at the same dose to all patients without additional drugs.

The limited number of patients and the absence of a control group are the limitations of our study. Our study was designed without a control group because it is ethically inappropriate to perform penile Doppler USG, which has risk of complications in the control groups consisting of healthy individuals and/or patients with mild/moderate ED. The strength of our study is that it is the only prospective clinical study in the literature in which, to our knowledge, the correlation of 2 biomarkers with hemodynamic parameters was investigated in patients who underwent penile Doppler USG with the correct indication.

There was no significant difference between the patients with normal hemodynamic parameters on penile Doppler USG and our other vasculogenic ED groups regarding Endocan and ADMA biomarker levels.

## 5. Conclusion

The findings obtained in this study suggest that Endocan and ADMA are not predictors of severe ED. There is a need for multicentral studies with larger patient populations to be conducted with different biomarkers that may have a higher predictive level in the future.

## Author contributions

**Conceptualization:** Binhan Kagan Aktas, Turker Soydas.

**Data curation:** Binhan Kagan Aktas.

**Formal analysis:** Emrah Gökay Ozgur.

**Investigation:** Binhan Kagan Aktas, Turker Soydas, Cagri Akpinar.

**Methodology:** Doruk Demirel, Binhan Kagan Aktas, Arzu Kosem, Turker Soydas, Cagri Akpinar.

**Project administration:** Doruk Demirel, Binhan Kagan Aktas

**Resources:** Doruk Demirel, Arzu Kosem, Turker Soydas, Onder Kayigil.

**Supervision:** Binhan Kagan Aktas, Turker Soydas, Cagri Akpinar, Cuneyt Ozden, Onder Kayigil.

**Validation:** Binhan Kagan Aktas.

**Visualization:** Binhan Kagan Aktas, Cagri Akpinar.

**Writing – original draft:** Doruk Demirel.

**Writing – review & editing:** Binhan Kagan Aktas.
